# Combination of Medical and Surgical Treatments for Masseter Hypertrophy

**DOI:** 10.1155/2018/7168472

**Published:** 2018-04-05

**Authors:** M. Ayhan, Sabri Cemil İşler, C. Kasapoglu

**Affiliations:** Faculty of Dentistry, Department of Oral and Maxillofacial Surgery, Istanbul University, Istanbul, Turkey

## Abstract

Masseter hypertrophy (MH) is one of the uncommon conditions that swelling can be seen in the angular mandibular region of the face. The etiology of MH includes several factors, and various treatment methods are mentioned in the literature. Botulinum toxin type A application is most commonly used for the treatment because of its less invasive feature. As a surgical method, some treatment alternatives that aim to reduce muscle mass or reshape the bone tissue in the angular region are considered. In this case report, a 21-year-old male patient with unilateral masseter hypertrophy on the right side is presented. After the patient was diagnosed with MH, botulinum toxin treatment in two sessions at one-month intervals was done. Since the reduction in muscle volume was not in satisfactory dimensions after the botulinum toxin application, the masseter was reduced on the right side through an intraoral approach. At the same time, bone enlargements on each side of the angulus mandibula were reshaped and smoothened through an extraoral retro mandibular approach. Clinical and radiographic evaluation of the patient revealed more aesthetic and symmetrical appearance in the regular controls.

## 1. Introduction

Masseter hypertrophy (MH) is an uncommon condition that can cause aesthetic and functional problems. Aesthetic problems consist of prominent masseter muscle in the face, rectangular face shape, and wide mandibular angle. Patients may suffer psychological issues due to an unattractive look [1]. Differential diagnosis requires clinical history and physical examination and may even include complementary imagination resources such as magnetic resonance (MR) and computed tomography (CT) scans to exclude other disorders. Differential diagnosis must consist of muscle tumors, salivary gland disorders, and intrinsic masseter myopathy. In some cases, patients may report signs and symptoms of well-localized pain [1, 2]. However, it is asymptomatic, and patients' chief complaint is about aesthetics. Moreover, masseteric musculature is inserted in the mandibular angle anatomically and can cause overdevelopment of these angles because of its traction forces [2]. The etiology of MH has been attributed to many factors such as tensions and clenching caused by emotional stress, chronic bruxism, masseteric hyperfunction, and parafunction. It is essential to make the differential diagnosis of head and neck mass, particularly unilateral mass located in the cheek. The possible underlying pathologic factors should be assessed carefully with detailed patient history and imaging techniques before deciding on treatment [3]. Treatment of MH is controversial. Varying degrees of success have been reported for some of the treatment options for MH which range from simple pharmacotherapy to more invasive surgery. Reduction of the masseter muscle, osteotomy, botulinum toxin, and splint therapy are options for managing this problem [2, 3]. Injection of botulinum toxin type A into the masseter muscle is considered as a less invasive modality and has been reported to be successfully used for cosmetic sculpting of the lower face [4]. Botulinum toxin type A (botulinum toxin) is a potent neurotoxin which is produced by the anaerobic organism *Clostridium botulinum* and when injected into a muscle causes interference with the neurotransmitter mechanism, producing selective paralysis and subsequent atrophy of the muscle [3–5]. Results showed the efficiency of botulinum in MH, but many times in MH concomitant with bone enlargement in the angulus; therefore, the best aesthetic results may be gained with manipulation of the bony structure. The traditional method of treatment for MH is the partial surgical excision of the masseter muscle and osteotomy of the mandibular angle region and reshaping the curvature of the bone under general anaesthesia [5]. The use of an intraoral approach was first suggested by Wood. He recommended the removal of bone enlargement of the mandibular angle without any masseter muscle manipulation. Tabrizi et al. advocated an intraoral approach including masseter muscle reduction and partially monocortical and bicortical osteotomy in the mandibular angle in the treatment of MH [3–6].

## 2. Case Presentation

**Figure 1:**
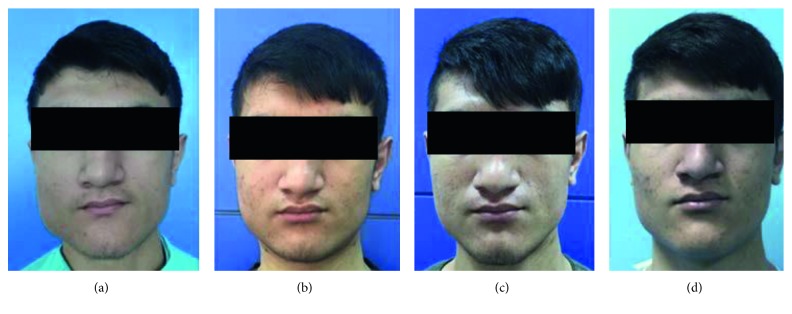
(a) Initial profile image; (b) profile image after one month of the first botulinum toxin application; (c) profile image after one month of the second botulinum toxin application; (d) profile image after six months of the surgery.

**Figure 2:**
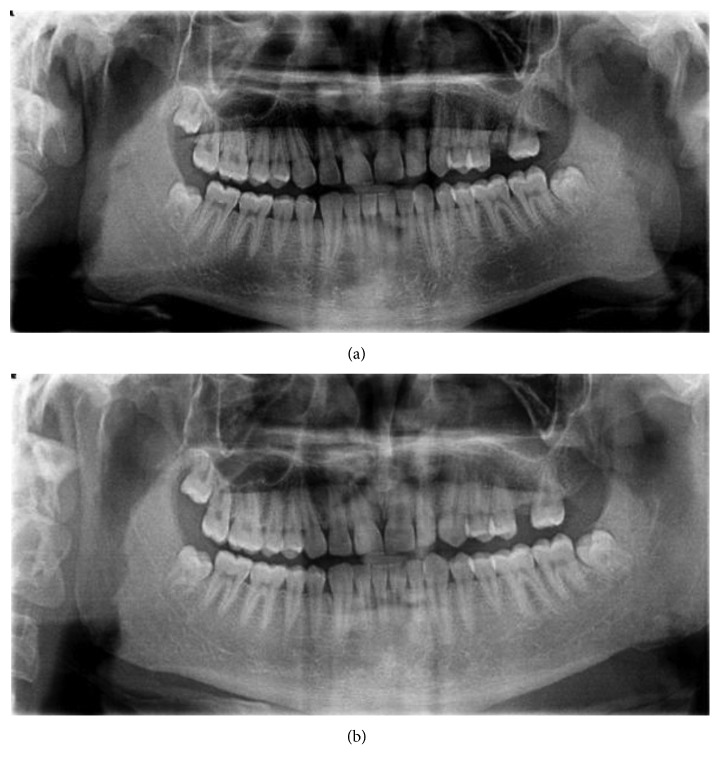
(a) Initial panoramic view; (b) panaromic view after six months of the surgery.

A 22-year-old male patient applied to our clinic for painless asymmetric swelling on the right side of the face for five years (Figure 1(a)). The history of the patient revealed that there are no parafunctional habits, functional and mouth opening limitation, bruxism, and trauma. And also, the masseteric region was nontender and normal in tone, and the temporomandibular joints and mandibular angulus region were not painful on palpation. The patient said that the only complaint was aesthetic and he wanted to have a more attractive facial appearance. Computed tomography, MR imagination, and panoramic radiographs were taken to make a differential diagnosis of MH. In MR examination, significant enlargement of the right masseter muscle compared to the left side was naturally detectable. There was also no pathological formation in the muscle. In CT and panoramic radiographs, reactive bone formation and significant asymmetry compared to the left side were observed in the mandibular angular region on the right side (Figure 2(a)). The patient was diagnosed with masseter hypertrophy. It was decided to apply botulinum toxin as the first step of the treatment.

### 2.1. Botulinum Toxin Application

Botulinum toxin type A (Botox; Allergan Inc., Irvine, CA) was supplied as a freeze-dried powder of 100 units and was reconstituted with 2 ml of sterile saline solution, giving a concentration of 100 units. Percutaneous intramuscular injection of botulinum toxin type A was performed to the hypertrophic muscle using 2 ml syringe with 25G needle. 75 units of botulinum toxin type A was injected equally into five points at the centre of the lower third of the masseter muscle (Figure 1(b)). Determining the number of injection points is based on our clinical experience and previous satisfactory results as injections are more homogenously located in the masseter muscle. A month later, an additional 60 units of botulinum toxin were applied to the muscle at the second visit. A decrease in the size of the masseter muscle was seen after one month of the application (Figure 1(c)). Within the six months' follow-up period, severe masseter muscle atrophy occurred, but although clinically significant atrophy has occurred, the patient was not entirely satisfied with his appearance. Thus the decision to perform surgery has made with permission of the patient and his family in order to reduce the volume of the right masseter muscle and soften the couture of the patients face.

### 2.2. Surgery

**Figure 3:**
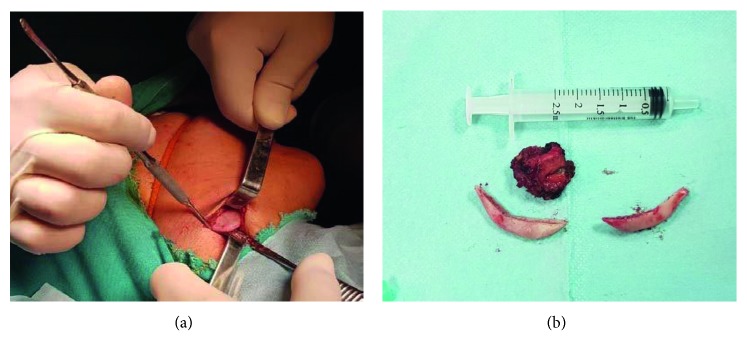
(a) Intraoperative view of the resected fragments of the right and left angulus of the mandible and 2/3 inner belly of the right masseter muscle; (b) extraoral submandibular approach.

**Figure 4:**
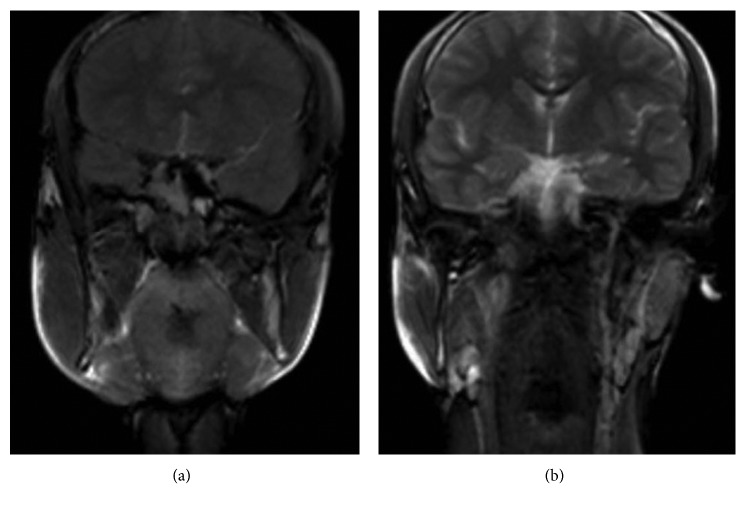
Pre-op (a) and post-op (b) MR views. Decreased volume of the right masseter muscle can be seen obviously.

The patient underwent surgery involving bilateral resection of mandibular angles and unilateral resection of the masseter muscle through intraoral and extraoral submandibular approaches. Under general anaesthesia, on the right side of the patient, an intraoral incision was made supraperiostally, slightly lateral to the external oblique line, and extended mandibular first molar region. The anterior portion of the masseter muscle was exposed, and the inner belly of the muscle was removed by the method described by Beckers [7]. The intraoral incision was closed with absorbable sutures. Next, by using the extraoral submandibular approach on both sides, after the skin incision was made 1.5 cm below the mandibular border, the platysma muscle and the superficial layer of the deep cervical fascia were sectioned, and with taking care of the marginal mandibular branch of the facial nerve, facial vein, and facial artery, the pterygomasseteric connection was reached. The pterygomasseteric connection was cut from the bottom of the mandible to the angular region. A bone cut was made on the lateral surface of the ramus via a piezoelectric surgery device on a curve shape line connecting a point about one-third height of the posterior border of the ramus and the anterior portion of the antegonial notch. Complete separation and removal of the segment from mandible were accomplished using a periosteum retractor (Figures 3(a) and 3(b)). The pterygomasseteric connection was closed with single absorbable suture, while the platysma was covered with absorbable continuous suture. Particular attention was shown to ensure that the underlying vascular structures and the mandibular nerve were not damaged during closure and the skin was closed. No drain was used in the surgery zone, and primary closure was performed. A pressure bandage and ice pack were applied for 72 hours. Antibiotic and analgesic therapy was prescribed. From MR views which were taken one year following the surgery, the decreased volume of the right masseter muscle can be seen apparently (Figures 4(a) and 4(b)). The patient was followed for one year without any problems (Figure 1(d)).

## 3. Discussion

There are many factors in the etiology of masseter hypertrophy, such as tensions and clenching caused by emotional stress and parafunctional habits. It is observed that none of the etiologic factors in the literature are present in our patient such as dental attrition, and therefore, we can refer to this condition as idiopathic masseter hypertrophy. Especially in the diagnosis of the clinical situation in unilateral hypertrophy, the differential diagnosis of head and neck soft tissue pathologies should be made [5, 7]. Before deciding on the treatment plan, MR and CT, thanks to their high imaging capacity, should be used to exclude possible pathologies such as muscle tumors, salivary gland disorders, parotid tumors, parotid inflammatory diseases, and intrinsic masseter myopathy. MR images of the patient in our case showed that the masseter muscle and surrounding soft tissues had a regular structure, but the right masseter muscle was significantly larger than the left side. Also, in panoramic radiographs of the patient, reactive bone formation and significant asymmetry compared to the left side were observed in response to the abnormal activity of the masseter muscle in the mandibular angular region on the right side. After the patient was diagnosed with MH, two options for treatment became prominent. One of them is the injection of botulinum toxin type A, and the other one is the respective surgery of the masseter muscle and angulus of the mandible. The use of botulinum toxin in the treatment of masseter hypertrophy is a frequently preferred treatment option since 1994. The minimally invasive nature of this procedure and the fact that the risk ratio is meager are the most important reasons why botulinum toxin is used for many years with success for cosmetic purposes [4, 8]. In the literature, botulinum toxin has been shown to cause muscle atrophy by blocking neurotransmission at the neurotransmitter junction. But, as the botulinum toxin activity declines over time, atrophic muscle tends to return to its former size, as neural conduction in the neurotransmitter junction will return to normal, so the injection should be repeated approximately every 4–8 months; however, there are various techniques and protocols for extending this lasting duration in the literature [8], which constitutes the biggest disadvantage of botulinum toxin treatment [4, 7]. On the other hand, surgical treatment, although providing more permanent results than botulinum toxin, has various complication risks such as hematoma formation, facial nerve paralysis, infection, mouth opening limitation, and appearance of scar [9]. After discussing the possible advantages and risks of both treatment options with the patient, botulinum toxin application was agreed as the first treatment option. 75 units of botulinum toxin was given at the first visit. One month later, at the second visit, 60 units of botulinum toxin were applied to the muscle. After six months, it was observed that the muscles had diminished to the initial position, but the symmetrical condition was still found to be unsatisfactory by the patient, and the surgical option was on the table. Some authors claim the partial removal of the masseter muscle is enough to correct MH. According to Beckers, the insertion of hypertrophic masseter muscle causes an abnormal growing mandibular angle. Other authors confirmed that, in order to reach satisfactory results, a mandibular angle resection should be performed [5, 7–10]. In our case, a more permanent and lighter facial contouring could be achieved compared to botulinum toxin treatment since the surgical procedure entails removing an appropriate amount of tissue from the mandibular angle and masseter muscle, and this can be observed when the patient's profile photographs, panoramic graphs, and MR images were taken before and after the surgery are compared (Figures 1, 2, and 4). In the literature, it is widely accepted that, to correct the MH, the resection of the inferior belly of the masseter muscle and angulus of the mandible should be performed with the intraoral approach [9–11]. However, despite the easy access to the inferior belly of the masseter muscle when using the intraoral approach, since the angulus of the mandibular is located in the thickest part of the operative area and the strong tension of the masseter muscle makes retraction very difficult, the surgeon's vision is limited in a sagittal direction [12, 13]. In our case, taking this limited access to the mandibular angular region when using the intraoral approach into account and since under these circumstances making the bone cut in the desired amount would be extremely difficult, the extraoral submandibular approach was chosen during the bone resection. The primary disadvantage of the extraoral approach is the risk of a scar on the face. The skin incision should be thrown parallel to the Langers lines and should be kept as short as possible to minimize this risk [13]. We have experienced no complication and scar with removal of the enlarged mandibular angle when using the combination of extraoral and intraoral approaches.

## 4. Conclusion

There is not yet a standard protocol in the literature about the treatment of masseter hypertrophy. When treatment is planned, the patient's expectations and physical findings should be evaluated thoroughly. In cases where masseter hypertrophy causes appositional changes in the bone, the success rate with botulinum toxin treatment alone is low, and the surgical option should be considered. Further experimental and clinical studies are necessary to estimate the success of surgical treatment in the short and long term.
